# Shaping humoral responses against mini-libraries of HIV env antigens via lipid nanoparticle vaccine delivery

**DOI:** 10.1186/1742-4690-9-S2-O63

**Published:** 2012-09-13

**Authors:** MC Hanson, J Mata-Fink, W Abraham, KD Wittrup, DJ Irvine

**Affiliations:** 1 Massachusetts Institute of Technology, Cambridge, MA, USA; 2 Ragon Institute of MGH, MIT and Harvard, Boston, MA, USA; 3 Howard Hughes Medical Institute, Chevy Chase, MD, USA

## Background

Humoral immune responses elicited by an HIV vaccine would ideally be comprised of durable high titers of broadly neutralizing antibodies. Importantly, recent studies of broadly neutralizing antibodies isolated from infected patients have suggested that high degrees of somatic hypermutation (SHM) are a common feature of antibodies with high potency and good breadth. Thus, a successful vaccine will likely require both immunogens capable of focusing the humoral response against conserved neutralizing epitopes and appropriate adjuvants/delivery systems capable of promoting elevated SHM and lasting responses against these epitopes.

## Methods

We generated a small library of gp120 mutants engineered to have diverse surface compositions but a conserved CD4 binding pocket recognized by the broadly neutralizing antibody VRC01. These gp120 mutants were linked to “stealth” liposomes via 2KDa PEG linkers. These lipid nanoparticles were simultaneously loaded with immunostimulatory adjuvant molecules such as MPLA (TLR4 agonist) or CpG (TLR9 agonist) to support differentiation of helper T-cells and promote avidity maturation of the antibody response. Mice were immunized repeatedly with stealth liposomes carrying unique gp120 mutants in each boost.

## Results

Stealth liposomes carrying TLR4 agonists (TLRa) promoted long-lived humoral responses against env antigens superior to traditional adjuvants such as alum, montanide, or soluble protein mixed with TLRa. Studies of liposome/antigen trafficking in vivo suggest these enhanced responses reflect efficient trafficking of these nanoparticle vectors to lymph nodes. Notably, this heterologous immunization strategy elicited anti-gp120 sera focused almost exclusively on the CD4 binding site and that competed with VRC01 for binding to gp120.

## Conclusion

This approach of combined immunogen design with effective multivalent, nanoparticle-based antigen delivery may provide a strategy to promote strong and long-lived neutralizing antibody responses against HIV.

This work was funded by the NIH (AI095109) and the Ragon Institute of MGH, MIT, and Harvard. DJI is an investigator of the Howard Hughes Medical Institute.

